# Time-resolved proteomic profile of *Amblyomma americanum* tick saliva during feeding

**DOI:** 10.1371/journal.pntd.0007758

**Published:** 2020-02-12

**Authors:** Tae Kwon Kim, Lucas Tirloni, Antônio F. M. Pinto, Jolene K. Diedrich, James J. Moresco, John R. Yates, Itabajara da Silva Vaz, Albert Mulenga

**Affiliations:** 1 Department of Veterinary Pathobiology, College of Veterinary Medicine, Texas A&M University, College Station, Texas, United States of America; 2 Centro de Biotecnologia, Universidade Federal do Rio Grande do Sul, Porto Alegre, Rio Grande do Sul, Brazil; 3 Foundation Peptide Biology Lab, Salk Institute for Biological Studies, La Jolla, Californai, United States of America; 4 Department of Molecular Medicine, The Scripps Research Institute, La Jolla, California, United States of America; 5 Mass Spectrometry Core, Salk Institute for Biological Studies, La Jolla, California, United States of America; 6 Faculdade de Veterinária, Universidade Federal do Rio Grande do Sul, Porto Alegre, Rio Grande do Sul, Brazil; University of Florida, UNITED STATES

## Abstract

*Amblyomma americanum* ticks transmit more than a third of human tick-borne disease (TBD) agents in the United States. Tick saliva proteins are critical to success of ticks as vectors of TBD agents, and thus might serve as targets in tick antigen-based vaccines to prevent TBD infections. We describe a systems biology approach to identify, by LC-MS/MS, saliva proteins (tick = 1182, rabbit = 335) that *A*. *americanum* ticks likely inject into the host every 24 h during the first 8 days of feeding, and towards the end of feeding. Searching against entries in GenBank grouped tick and rabbit proteins into 27 and 25 functional categories. Aside from housekeeping-like proteins, majority of tick saliva proteins belong to the tick-specific (no homology to non-tick organisms: 32%), protease inhibitors (13%), proteases (8%), glycine-rich proteins (6%) and lipocalins (4%) categories. Global secretion dynamics analysis suggests that majority (74%) of proteins in this study are associated with regulating initial tick feeding functions and transmission of pathogens as they are secreted within 24–48 h of tick attachment. Comparative analysis of the *A*. *americanum* tick saliva proteome to five other tick saliva proteomes identified 284 conserved tick saliva proteins: we speculate that these regulate critical tick feeding functions and might serve as tick vaccine antigens. We discuss our findings in the context of understanding *A*. *americanum* tick feeding physiology as a means through which we can find effective targets for a vaccine against tick feeding.

## Author summary

The lone star tick, *Amblyomma americanum*, is a medically important species in US that transmits 5 of the 16 reported tick-borne disease agents. Most recently, bites of this tick were associated with red meat allergies in humans. Vaccination of animals against tick feeding has been shown to be a sustainable and an effective alternative to current acaricide based tick control method which has several limitations. The pre-requisite to tick vaccine development is to understand the molecular basis of tick feeding physiology. Toward this goal, this study has identified proteins that *A*. *americanum* ticks inject into the host at different phases of its feeding cycle. This data set has identified proteins that *A*. *americanum* inject into the host within 24–48 h of feeding before it starts to transmit pathogens. Of high importance, we identified 284 proteins that are present in saliva of other tick species, which we suspect regulate important role(s) in tick feeding success and might represent rich source target antigens for a tick vaccine. Overall, this study provides a foundation to understand the molecular mechanisms regulating tick feeding physiology.

## Introduction

Ticks and tick-borne diseases (TBDs) have been on the rise and have greatly impacted human and veterinary medicine. Ticks have gained the attention in public health policy with a recent publication that advocated for One Health solutions listing 17 human TBDs among sources of human health concerns [1]. Moreover, the dramatic rise related to ticks and TBDs have caught the attention of United States (US) lawmakers, as shown in the 21^st^ Century Cures Act of 2016, which created the TBD Working Group. Under the Cures Act, the TBD Working Group was tasked with evaluating the impact of TBDs and required research to find solutions (https://www.hhs.gov/ash/advisory-committees/tickbornedisease/index.html). Likewise, six of the 23 human vector-borne diseases that are listed by the World Health Organization are tick-borne that include Crimean-Congo haemorrhagic fever, Lyme disease, relapsing fever, rickettsial diseases (spotted fever and Q fever), tick-borne encephalitis, and tularemia (http://www.who.int/news-room/fact-sheets/detail/vector-borne-diseases). In the US, *Amblyomma americanum*, the lone star tick is among one of the tick species of medical and veterinary health significance.

*A*. *americanum* is a geographically expanding tick species [2] that is involved in transmission of multiple human and animal disease agents. In public health, *A*. *americanum* is the principal vector for *Ehrlichia chaffensis*, the causative agent of human monocytic ehrlichiosis [3], and *E*. *ewingii*, which also causes ehrlichiosis, referred to as human granulocytic ehrlichiosis [4–6]. This tick also transmits *Francisella tularensis*, the causative agent for tularemia [7, 8], a yet to be described disease agent, suspected as *Borrelia lonestari*, which causes Lyme disease-like symptoms referred to as southern tick-associated rash illness (STARI) [9, 10] and also an *E*. *ruminantium-*like organism referred to as the Panola Mountain *Ehrlichia* (PME) [11]. There is also evidence that *A*. *americanum* may transmit *Rickettsia amblyommii*, *R*. *rickettsia*, *and R*. *parkeri*, the causative agents to rickettsiosis to humans [12, 13]. This tick has also been reported to transmit the Heartland and Bourbon viruses to humans [14, 15]. Most recently, this tick has been shown to be responsible for causing an α-gal allergy or mammalian meat allergy (MMA) in humans upon tick bite [16]. In veterinary health, *A*. *americanum* transmits *Theileria cervi* to deer [17], and *E*. *ewingii* to dogs [18]. There are reports of mortality in deer fawns that were attributed to a combination of heavy *A*. *americanum* infestation and *T*. *cervi* infections [19]. In livestock production, heavy infestations were thought to cause low productivity in cattle [20, 21]. In the Southern US, *A*. *americanum* appears to be the most dominant tick species that bite humans, which has been reported to be responsible for 83% of human tick infestations [22].

The success of ticks as pests and vectors of TBD agents is facilitated by secreted tick salivary proteins that are injected into the host to regulate the tick’s evasion of host defense [23]. There is evidence that repeatedly infested animals develop immunity against tick saliva proteins and are protected against TBD transmissions such as *Francisella tularensis* [24], *B*. *burgdorferi* [25–27], *Babesia* spp. [28], Thogoto virus [29], tick-borne encephalitis virus [30] and *T*. *parva bovis* [31]. Therefore, identification of tick saliva proteins that ticks inject into the host during feeding might lead to development of tick saliva protein-based vaccines to prevent TBD infections.

The goal of this study was to utilize systems biology approach to identify proteins that *A*. *americanum* ticks injects during feeding. This study builds upon our recent findings that identified *Ixodes scapularis* tick saliva proteins that are secreted every 24 h during first five days of feeding [32], partial and replete fed *Rhipicephalus microplus* [33], and replete fed adult and nymph *Haemaphysalis longicornis* [34]. Others have reported proteins in saliva of replete fed *R*. *sanguineus* [35] and three and five day fed *Dermacentor andersoni* [36]. A related study reported proteins in *Ornithodoros moubata* (soft tick) identified from saliva collected after four months from feeding [37]. Most recently, the saliva proteomes of unfed *I*. *scapularis* and *A*. *americanum* exposed to different hosts have been identified [38]. In this study, we report proteins that *A*. *americanum* ticks sequentially inject into the host every 24 h during feeding. Comparison of the *A*. *americanum* tick saliva proteome in this study with other saliva proteomes of other tick species allowed us to identify tick saliva proteins that are likely utilized by multiple tick species to regulate feeding, and these might represent potential antigens for anti-tick vaccine development.

## Materials and methods

### Ethics statement

All experiments were done according to the animal use protocol approved by Texas A&M University Institutional Animal Care and Use Committee (IACUC) (AUP 2011–207 and 2011–189) that meets all federal requirements, as defined in the Animal Welfare Act (AWA), the Public Health Service Policy (PHS), and the Humane Care and Use of Laboratory Animals.

### *A*. *americanum* tick saliva collection

*A*. *americanum* ticks were purchased from the tick rearing facility at Oklahoma State University (Stillwater, OK, USA). Routinely, ticks were fed on rabbits as previously described [39, 40]. Ticks were restricted to feed on the outer part of the ear of New Zealand rabbits with orthopedic stockinet’s glued with Kamar adhesive (Kamar Products Inc., Zionsville, IN, USA). To stimulate female *A*. *americanum* ticks to attach onto the host to start feeding and to be inseminated to complete the feeding process, male ticks (15 per ear) were pre-fed for three days prior to placing female ticks onto rabbit ears to feed. A total of 50 female *A*. *americanum* ticks (25 per ear) were placed into tick containment apparatus on each of the three rabbits and allowed to attach.

Saliva of female *A*. *americanum* tick was collected as previously described [32, 33]. Saliva was collected and pooled from 30 ticks fed for 24, 48, 72, 96, 120, 144, 168, and 192 h, respectively, ten ticks for apparently engorged but not detached from the host (BD), and six ticks for replete fed and spontaneously detached from the host (SD). Tick saliva was collected every 15–30 min intervals for a period of approximately 4 h at room temperature from ticks that were previously injected with 1–3 μL of 2% pilocarpine hydrochloride in phosphate buffered saline (PBS, pH 7.4) as published by our group [32, 38]. Protein quantification was routinely done using the Pierce BCA (bicinchoninic acid assay) Protein Assay Kit (Thermo Fisher Scientific, Waltham, MA, USA).

### Identification of *A*. *americanum* tick saliva proteins by LC-MS/MS

*A*. *americanum* tick saliva proteins were identified from an in-solution preparation method using LC-MS/MS as previously described [32–34]. Approximately 2 μg of total tick saliva proteins (in triplicate runs) per feeding time point (24, 36, 48, 72, 96, 120, 144, 168, 192 h fed, BD, and SD) were diluted in 8 M urea/0.1 M Tris (pH 8.5), reduced with 5 mM Tris (2-carboxyethyl) phosphine hydrochloride (TCEP, Sigma-Aldrich, St Louis, MO, USA), and alkylated with 25 mM iodoaceamide (Sigma-Aldrich). Proteins were digested overnight at 37°C in 2 M urea/0.1M Tris pH 8.5, 1 mM CaCl_2_ with trypsin (Promega, Madison, WI, USA) with a final ratio of 1:20 (enzyme:substrate). Digestion reactions, in a final concentration of 0.15 μg/mL (per technical replicate), were quenched with formic acid (5% final concentration) and centrifuged for debris removal before peptide mixtures were analyzed by nanoflow liquid chromatography mass spectrometry using an Easy NanoLC II and a Q Exactive mass spectrometer (Thermo Fisher Scientific). The mass spectrometry proteomics data have been deposited into ProteomeXchange Consortium via the PRIDE [41] partner repository with the dataset identifier PXD014844.

### Database searching of tandem mass spectra

Proteins in *A*. *americanum* tick saliva were identified according to the previously described pipeline [32–34]. To prepare the protein database used for protein identification, we extracted the coding sequences (CDS) from *A*. *americanum* transcriptomes that were assembled from Illumina sequence reads (BioProject accession # PRJNA226980) [42] using an automated pipeline in Visual Basic (Microsoft, Redmond, Washington, USA) provided Dr. Jose M. Ribeiro (NIH), based on similarities to known proteins [43]. Contigs from the assembled *A*. *americanum* transcriptome were used to identify open reading frames (ORFs) that were larger than 50 amino acids in all six frames. For functional annotation, the identified ORFs were subjected to blastp using several amino acid sequence databases downloaded from NCBI (non-redundant [nr] Acari and refseq-invertebrate), Uniprot (nr-Acari), MEROPS database [44], the GeneOntology (GO) FASTA subset [45] and the conserved domains database (CDD) of NCBI [46] containing the COG [47], PFAM [48], and SMART motifs [49]. Outputs from the blast searches were used in the classifier program in Dr. Ribeiro's visual basic program [43] to functionally categorize the identified proteins based on the best match from among all the blast screens. The functionally annotated proteins were manually validated. As a false-discovery approach to identify transcripts related to hosts, we searched the ORFs against the nr-databases from NCBI for rabbit, mouse, rat, goat, sheep, cow, monkey, and humans. CDS were extracted from blastp searches that matched with 70% identity and e-value of 1e^-40^. To remove redundancies, CD-HIT [50] was used to remove sequences at 98% identity. The extracted CDS (n = 110,587) were concatenated with *Oryctolagus cuniculus* from Uniprot (www.uniprot.org) (n = 21,148) and reverse sequences of all entries were used to identify peptides from tandem mass spectra.

Proteins were identified by first extracting the tandem mass spectra from Thermo RAW files using RawExtract 1.9.9.2 [51] and then searching against the protein database (described above) using ProLuCID in the Integrated Proteomics Pipeline Ver.5.0.1 [52]. At least two peptide matches were required to be considered a protein hit. A cutoff score was established to accept a protein false discovery rate (FDR) of 1% based on the number of decoys. Additionally, a minimum sequence length of six residues per peptide was required. Results were post processed to only accept PSMs with <10ppm precursor mass error. Finally, the protein matches from each sampled time points were concatenated into one file using the Identification Compare (IDcompare) program on IP2- Integrated Proteomics Pipeline Ver.5.0.1 [52].

### Relative abundance and graphical visualization of secretion dynamics of *A*. *americanum* tick saliva proteins

Relative abundance and secretion dynamics were determined as described [32] using normalized spectral abundance factors (NSAF) that were validated as reliable in a label-free relative quantification approach [53–55]. For each functional category or individual protein, NSAF was expressed as a percent (%) of total NSAF for that time point. Percent NSAF values were normalized using Z-score statistics using the formula $Z=\frac{X-\mu }{\sigma }$, where *Z i*s the Z-score, *X* is the NSAF for each protein per time point, *μ* is the mean throughout time points, *σ* is the standard deviation throughout time points. Normalized percent NSAF values were used to generate heat maps using the heatmap2 function from the gplots library in R [56]. Secretion dynamics (low to high abundance) were used to assemble clusters on heat maps.

### Identification of *A*. *americanum* saliva proteins found in saliva of other tick species

*A*. *americanum* tick saliva proteins in this study were searched against published tick saliva proteomes of *R*. *microplus* [32], *I*. *scapularis* [33], *H*. *longicornis* [34], *R*. *sanguineus* [35], *D*. *andersoni* [36], and *O*. *moubata* [37] using local BLASTp analysis. Databases of protein sequences reported for each tick saliva proteome were extracted from NCBI or Uniprot and screened by BLASTp using the *A*. *americanum* saliva proteome (from this study) as the query. Protein matches ≥70% identity was reported.

## Results and discussion

Protein profile and abundance changes every 24 h during *A*. *americanum* tick feeding Previous studies have demonstrated that the protein profile and abundance in salivary glands of female *A*. *americanum* is dynamic and changes during the course of tick feeding [57]. However, a limitation to the previous study was that it did not inform which salivary gland proteins were secreted during feeding. To attempt at capturing changes in tick saliva protein profiles, we successfully used pilocarpine to induce and collect saliva from *A*. *americanum* ticks every 24 h during the first eight days of tick feeding as we all as from ticks that had engorged but had not detached, and replete fed ticks [32, 58]. In early feeding stages (24–72 h), *A*. *americanum* tick saliva was observed as a white flake that accumulated on the mouthparts over time and was collected every 15–30 min for 4 h by washing the mouthparts with sterile phosphate buffered saline. Tick saliva was more evident after 72 h of feeding, observed as droplets of liquid forming at the mouthparts.

Saliva collected from ticks that had fed for 24, 48, 72, 96, 120, 144, 168, and 192 h as well as ticks that were apparently engorged but were not detached from the host (BD) and replete fed (SD) was subjected to LC-MS/MS analysis for protein identification. Peptide mass spectra were searched against the combined database (described above) using the ProLuCID search engine [52]. We identified 450, 540, 419, 441, 332, 529, 478, 536, 312, and 325 tick proteins in the 10 different saliva samples (S1 Table). Similarly, we respectively identified 127, 130, 115, 147, 112, 140, 199, 198, 78, and 282 as rabbit proteins (S1 Table). The identification of 1182 tick and 335 rabbit unique proteins in tick saliva demonstrates the complexity of tick and host interactions.

### Tick and rabbit proteins in *A*. *americanum* tick saliva are annotated in multiple functional categories

With redundancy removed at 98% amino acid identity levels, we have identified 1182 tick and 335 rabbit unique proteins, which we have categorized into a respective 27 (Tables 1 and 2) and 25 (Tables 3 and 4) functional categories. Data in Tables 1–4 summarizes total number of proteins (protein count) and cumulative relative abundance based on normalized spectral abundance factor (NSAF, the index for relative protein abundance [53–55]) for each functional category. Please note that Tables 1 and 3 summarizes respective tick and rabbit proteins that were identified in 24 to 120 h saliva, while Tables 2 and 4 has proteins that were identified in 144–192 h, BD and SD saliva.

**Table 1:** Numbers and cumulative relative abundance of tick protein classes in *Amblyomma americanum* saliva during 24–120 h of feeding (NSAF = normalized spectral abundance factor).

	Feeding Time Point									
	24 h		48 h		72 h		96 h		120 h	
Classification	Protein Count	NSAF (%)	Protein Count	NSAF (%)	Protein Count	NSAF (%)	Protein Count	NSAF (%)	Protein Count	NSAF (%)
antimicrobial	6	4.20	6	3.30	5	6.01	5	6.15	5	6.73
cytoskeletal	42	4.77	16	1.32	25	2.13	14	1.86	6	0.71
detoxification	19	2.16	15	0.71	12	0.72	15	0.89	3	0.18
evasin	0	0.00	6	0.74	2	0.24	6	0.68	7	1.67
extracellular matrix	20	1.81	11	1.20	17	1.48	11	0.64	6	0.37
glycine rich	23	2.41	43	5.81	44	9.82	41	4.86	27	6.83
heme/iron binding	16	20.20	16	7.48	15	15.27	16	12.35	9	5.81
immune-related	11	2.96	9	2.28	8	4.12	9	4.47	7	3.54
ixodegrin	1	0.46	5	0.82	1	0.84	2	0.47	2	0.62
lipocalin	8	0.74	17	2.90	2	0.55	6	1.01	3	4.39
metabolism, amino acid	3	0.08	0	0.00	1	0.02	0	0.00	0	0.00
metabolism, carbohydrate	11	0.57	7	0.22	8	0.36	9	0.77	3	0.06
metabolism, energy	18	1.22	17	1.54	13	1.48	10	0.94	2	0.04
metabolism, lipid	14	1.07	19	1.14	18	1.19	14	1.33	8	0.57
metabolism, nucleic acid	7	2.48	18	1.99	9	0.97	9	2.19	13	1.22
mucin	3	0.21	5	0.53	4	0.54	6	0.96	4	0.27
nuclear regulation	7	0.39	7	0.45	8	1.04	7	1.24	7	1.00
protease	17	0.72	25	1.07	20	0.97	25	1.46	27	4.08
protease inhibitor	55	22.28	76	21.14	52	11.10	64	15.33	66	20.32
proteasome machinery	8	1.57	6	0.50	6	1.29	6	1.50	6	1.18
protein modification	16	0.59	6	0.14	16	0.84	7	0.22	5	0.13
protein synthesis	6	0.16	2	0.05	4	0.14	2	0.06	0	0.00
signal transduction	14	3.57	11	4.74	3	0.39	7	2.01	8	1.80
tick specific proteins	110	23.99	188	39.44	118	37.88	140	37.87	105	38.28
transcription machinery	4	0.24	1	0.03	3	0.18	3	0.11	0	0.00
transporter/ receptor	10	1.12	6	0.42	4	0.37	7	0.62	3	0.19
transposon element	1	0.02	2	0.03	1	0.05	0	0.00	0	0.00
Total	450	100.00	540	100.00	419	100.00	441	100.00	332	100.00

**Table 2:** Numbers and cumulative relative abundance of tick protein classes in *Amblyomma americanum* saliva during 144 to completion of feeding (NSAF = normalized spectral abundance factor).

	Feeding Time Point									
	144 h		168 h		192 h		BD		SD	
Classification	Protein Count	NSAF (%)	Protein Count	NSAF (%)	Protein Count	NSAF (%)	Protein Count	NSAF (%)	Protein Count	NSAF (%)
antimicrobial	4	4.31	4	2.87	3	2.98	3	5.04	3	1.49
cytoskeletal	38	3.43	11	0.98	16	1.23	3	0.39	14	1.42
detoxification	21	1.71	11	0.67	18	1.13	7	0.79	12	0.55
evasin	5	1.54	7	3.40	6	3.39	1	0.83	5	2.86
extracellular matrix	18	1.61	7	0.38	19	0.85	14	1.75	0	0.00
glycine rich	14	1.03	19	2.00	31	2.79	9	0.47	8	0.56
heme/iron binding	17	22.96	14	7.31	16	12.38	17	26.07	10	7.23
immune-related	14	3.76	9	2.37	14	3.09	12	4.65	7	1.66
ixodegrin	1	0.58	1	0.20	1	0.62	2	0.78	0	0.00
lipocalin	8	1.11	24	6.57	20	5.69	20	4.07	26	14.76
metabolism, amino acid	4	0.07	2	0.07	1	0.04	0	0.00	0	0.00
metabolism, carbohydrate	22	1.30	4	0.12	14	0.90	14	2.56	5	0.17
metabolism, energy	13	0.81	9	0.34	10	0.50	2	0.30	6	0.15
metabolism, lipid	16	0.95	5	0.39	16	1.01	12	1.29	3	0.39
metabolism, nucleic acid	14	1.80	10	0.93	16	1.72	3	1.11	8	1.21
mucin	7	0.33	3	0.13	9	0.33	3	0.56	0	0.00
nuclear regulation	10	0.74	8	1.49	8	1.02	1	0.26	11	2.15
protease	40	3.37	38	4.57	43	4.21	36	2.93	36	7.70
protease inhibitor	83	17.41	81	15.82	78	14.79	59	19.17	39	17.08
proteasome machinery	7	0.87	6	1.09	6	0.87	4	0.11	6	1.68
protein modification	18	0.58	10	0.26	13	0.38	0	0.00	11	0.44
protein synthesis	2	0.03	2	0.06	5	0.15	0	0.00	2	0.08
signal transduction	14	3.11	10	1.49	9	2.43	7	1.89	5	0.53
tick specific proteins	127	25.15	175	46.18	157	36.86	77	23.84	103	37.75
transcription machinery	2	0.04	1	0.02	1	0.01	0	0.00	1	0.02
transporter/ receptor	10	1.38	6	0.26	6	0.62	6	1.14	3	0.07
transposon element	0	0.00	1	0.04	0	0.00	0	0.00	1	0.02
Total	529	100.00	478	100.00	536	100.00	312	100.00	325	100.00

**Table 3:** Numbers and cumulative relative abundance of rabbit protein classes in *Amblyomma americanum* saliva during 24–120 h of feeding (NSAF = normalized spectral abundance factor).

	Feeding Time Point									
	24 h		48 h		72 h		96 h		120 h	
Classification	Protein Count	NSAF (%)	Protein Count	NSAF (%)	Protein Count	NSAF (%)	Protein Count	NSAF (%)	Protein Count	NSAF (%)
antimicrobial	3	0.00134357	4	0.00192864	3	0.00171397	5	0.0086771	6	0.00763598
antioxidant	2	0.00042155	1	7.576E-05	1	0.00035322	0	0	0	0
cytoskeletal	19	0.01798265	14	0.01629188	11	0.00953933	12	0.01698913	7	0.01026072
extracellular matrix	3	0.00049492	1	0.000267	1	0.0000291	2	0.0001466	2	0.0003605
fibrinogen	3	0.00022576	4	0.00017475	0	0	1	6.7612E-05	2	0.00022745
globin/ RBC	20	0.08991802	18	0.02390767	12	0.01107268	15	0.02658361	15	0.02722383
heme/Iron binding	5	0.01948816	7	0.01314713	2	0.00443612	7	0.00916951	4	0.00596773
immunity related	9	0.00268497	7	0.0023492	5	0.00211006	8	0.00712898	15	0.01097912
keratin	17	0.00337019	32	0.01031924	35	0.02503904	41	0.04692297	27	0.03080678
lipocalin	0	0	1	0.00051272	1	0.00052663	1	0.00283525	1	0.00167895
metabolism, amino acid	0	0	0	0	0	0	0	0	0	0
metabolism, carbohydrates	5	0.00133936	4	0.00037672	5	0.00064097	5	0.00074812	3	0.00033307
metabolism, energy	1	2.8207E-05	0	0	0	0	3	0.00071594	1	0.00018482
metabolism, lipid	0	0	0	0	0	0	2	0.00093604	2	0.00138574
metabolism, nucleic acids	0	0	0	0	0	0	0	0	0	0
nuclear regulation	8	0.00325694	16	0.00871704	15	0.01681692	18	0.01938734	15	0.01609353
protease	0	0	1	0.00013596	0	0	2	0.00146001	1	4.2416E-05
protease Inhibitors	8	0.0025413	5	0.00116042	1	0.0001394	5	0.00178596	1	0.00071414
protein export	0	0	2	0.00017604	2	0.00115523	3	0.00209215	3	0.00247782
protein modification	7	0.00102993	3	0.0003923	7	0.00221869	6	0.00095378	4	0.00051132
protein synthesis	4	0.00135166	2	0.0001756	4	0.00122153	4	0.0006344	0	0
proteasome machinery	2	0.00600273	2	0.0021349	2	0.00578655	2	0.0056957	2	0.00490593
signal transduction	9	0.00264391	1	0.00047693	7	0.00264375	1	0.00089234	1	0.00035228
transporter/ receptor	2	0.00013431	5	0.00049163	1	3.5648E-05	4	0.00061835	0	0
transcription machinery	0	0	0	0	0	0	0	0	0	0
Total	127	0.15425811	130	0.08321153	115	0.08547884	147	0.15444091	112	0.12214214

**Table 4:** Numbers and cumulative relative abundance of rabbit protein classes in *Amblyomma americanum* saliva during 144 to completion of feeding (NSAF = normalized spectral abundance factor).

	Feeding Time Point									
	144 h		168 h		192 h		BD		SD	
Classification	Protein Count	NSAF (%)	Protein Count	NSAF (%)	Protein Count	NSAF (%)	Protein Count	NSAF (%)	Protein Count	NSAF (%)
antimicrobial	8	0.007565	9	0.018337	9	0.015212	6	0.004394	8	0.015088
antioxidant	3	0.000677	2	0.00071	3	0.000384	0	0	4	0.000927
cytoskeletal	20	0.019936	29	0.014844	26	0.019163	6	0.011922	55	0.031601
extracellular matrix	1	3.63E-05	0	0	1	2.58E-05	1	8.41E-05	3	0.000973
fibrinogen	1	0.00011	5	0.002512	5	0.001638	0	0	5	0.001423
globin/ RBC	18	0.105112	19	0.100601	18	0.067832	16	0.052725	21	0.109247
heme/Iron binding	3	0.005258	9	0.011255	7	0.011786	3	0.005854	9	0.015765
immunity related	15	0.007953	23	0.02019	17	0.014264	5	0.002246	23	0.025243
keratin	20	0.010315	34	0.012893	37	0.021782	21	0.010099	40	0.045599
lipocalin	1	0.002122	1	0.004206	1	0.00311	1	0.001514	1	0.00534
metabolism, amino acid	0	0	1	0.000201	0	0	0	0	1	3.83E-05
metabolism, carbohydrates	5	0.001337	9	0.001486	7	0.001096	2	0.000114	7	0.001634
metabolism, energy	2	0.000319	2	0.000144	2	0.000364	0	0	6	0.000535
metabolism, lipid	0	0	0	0	0	0	0	0	2	0.000229
metabolism, nucleic acids	1	0.000108	4	0.000989	0	0	0	0	0	0
nuclear regulation	19	0.013472	20	0.028935	24	0.022492	10	0.008565	26	0.032846
protease	0	0	4	0.000876	1	9.16E-05	0	0	6	0.00066
protease Inhibitors	1	0.000202	3	0.000967	2	0.000151	0	0	7	0.00203
protein export	3	0.001588	4	0.004882	5	0.003771	2	0.00077	4	0.006886
protein modification	6	0.000988	7	0.000754	12	0.001567	0	0	16	0.002522
protein synthesis	0	0	4	0.00057	4	0.00084	0	0	4	0.000866
proteasome machinery	2	0.003209	2	0.003649	2	0.003418	2	0.000584	2	0.005294
signal transduction	8	0.003392	5	0.001237	9	0.001945	0	0	12	0.003857
transporter/ receptor	3	0.000381	1	6.81E-05	4	0.000521	3	0.000589	13	0.00182
transcription machinery	0	0	2	6.16E-05	2	6.68E-05	0	0	7	0.000636
Total	140	0.184079	199	0.230369	198	0.191521	78	0.099459	282	0.311061

Overall, majority of the identified proteins are tick specific proteins (did not match to proteins of non-tick organisms) of unknown function (32%), followed by protease inhibitors (PI) (13%), proteases (8%), and glycine-rich proteins (6%). Notable protein categories that were ≤ 5% include cytoskeletal, lipocalin, antioxidant/detoxification, extracellular matrix, immune related, heme/iron-binding, mucins, evasins, antimicrobials, and ixodegrins (Tables 1 and 2, S1 Table). For rabbit proteins, the majority are categorized as cytoskeletal (19%), followed by keratin (13%), nuclear regulation (8%), immunity-related (8%), globin/RBC degradation (6%), and protein categories that were ≤ 5% include antimicrobials, heme/iron-binding, protease inhibitors, proteases, extracellular matrix, antioxidant/detoxification, fibrinogen and lipocalin (Tables 3 and 4, S1 Table).

### The most abundant category of *A*. *americanum* tick saliva proteins is tick-specific proteins of unknown function

**Fig 1:**
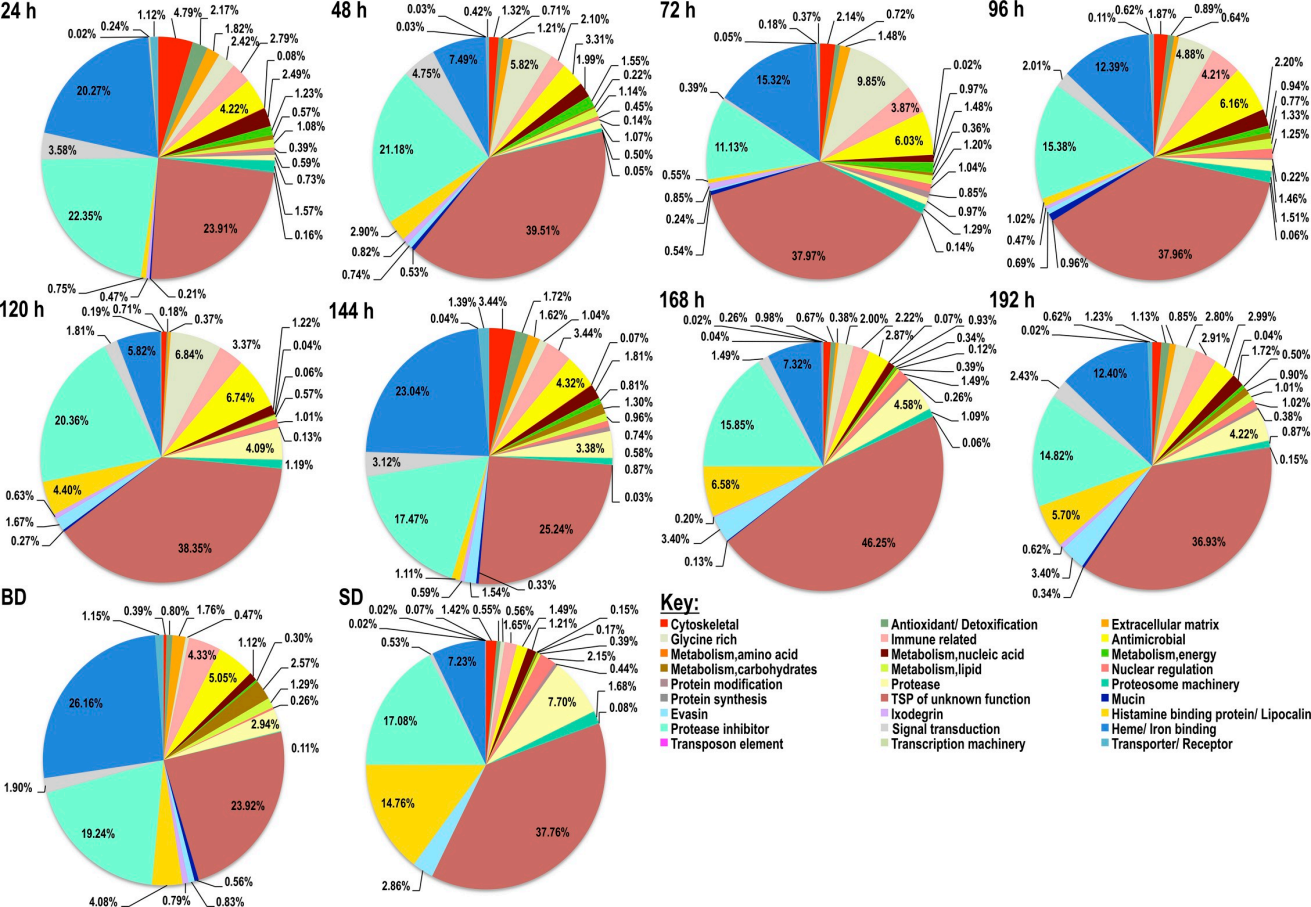
Tick saliva proteins composition in *Amblyomma americanum* tick saliva every 24 h during feeding. Pilocarpine induced *A*. *americanum* tick saliva was subjected to LC-MS/MS sequencing using the “In-Solution” digestion approach as described in materials and methods. Cumulative normalized spectral abundance factors (NSAF) values as index for relative abundance of detected tick saliva proteins are presented for each functional class. Please note the labels are oriented horizontally and read left to right starting from cytoskeletal proteins (red), antioxidant/detoxification (green), extracellular matrix (orange), then back to glycine rich proteins (light-tan), immune related (pink) and continues this pattern.

**Fig 2:**
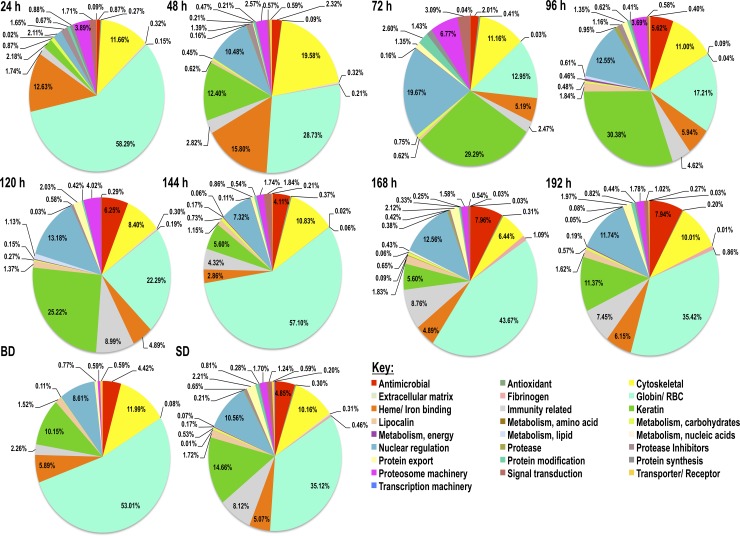
Rabbit host proteins composition in *Amblyomma americanum* tick saliva every 24 h during feeding. Pilocarpine induced *A*. *americanum* tick saliva was subjected to LC-MS/MS sequencing using the “In-Solution” digestion approach as described in materials and methods. Cumulative normalized spectral abundance factors (NSAF) values as index for relative abundance of detected rabbit host proteins are presented for each functional class. Please note the labels are oriented horizontally and read left to right starting from antimicrobial proteins (red), antioxidant/detoxification (green), cytoskeletal (yellow), then back to globin/RBC (light-green), fibrinogen (pink) and continues this pattern.

To get insights into relative abundance of each functional category, sum totals of NSAF values for each functional category are summarized in Figs 1 and 2. As shown in Fig 1, tick-specific secreted saliva proteins of unknown function (TSP), protease inhibitors (PI), and heme/iron binding proteins are the most abundant ranging from a respective 24–46%, 11–22%, and 6–26% during feeding (24-192h). Other protein categories at ≤ 10% in abundance include glycine-rich proteins, antimicrobial peptides, evasins, and proteases. For rabbit proteins in *A*. *americanum* tick saliva, the most predominant functional category was hemoglobin/red blood cell products (RBC) (13–58%) followed by cytoskeletal (6–20%), heme/iron binding (5–16%), keratin (2–30%), and nuclear regulation (2–20%) (Fig 2). It is notable that rabbit functional categories related to immunity, antimicrobial peptides, protease inhibitors and proteases were abundant at ≤8% throughout feeding.

The finding that the majority of proteins identified in this study are of unknown function is not unique to *A*. *americanum* tick saliva, it is consistent with findings in saliva of *I*. *scapularis* [32] and tick salivary gland transcriptomics [59–61]. This is potentially a reflection of how little information exists on the molecular basis of tick feeding physiology.

### Majority of *A*. *americanum* tick saliva proteins are associated with early stage tick-feeding processes

**Fig 3:**
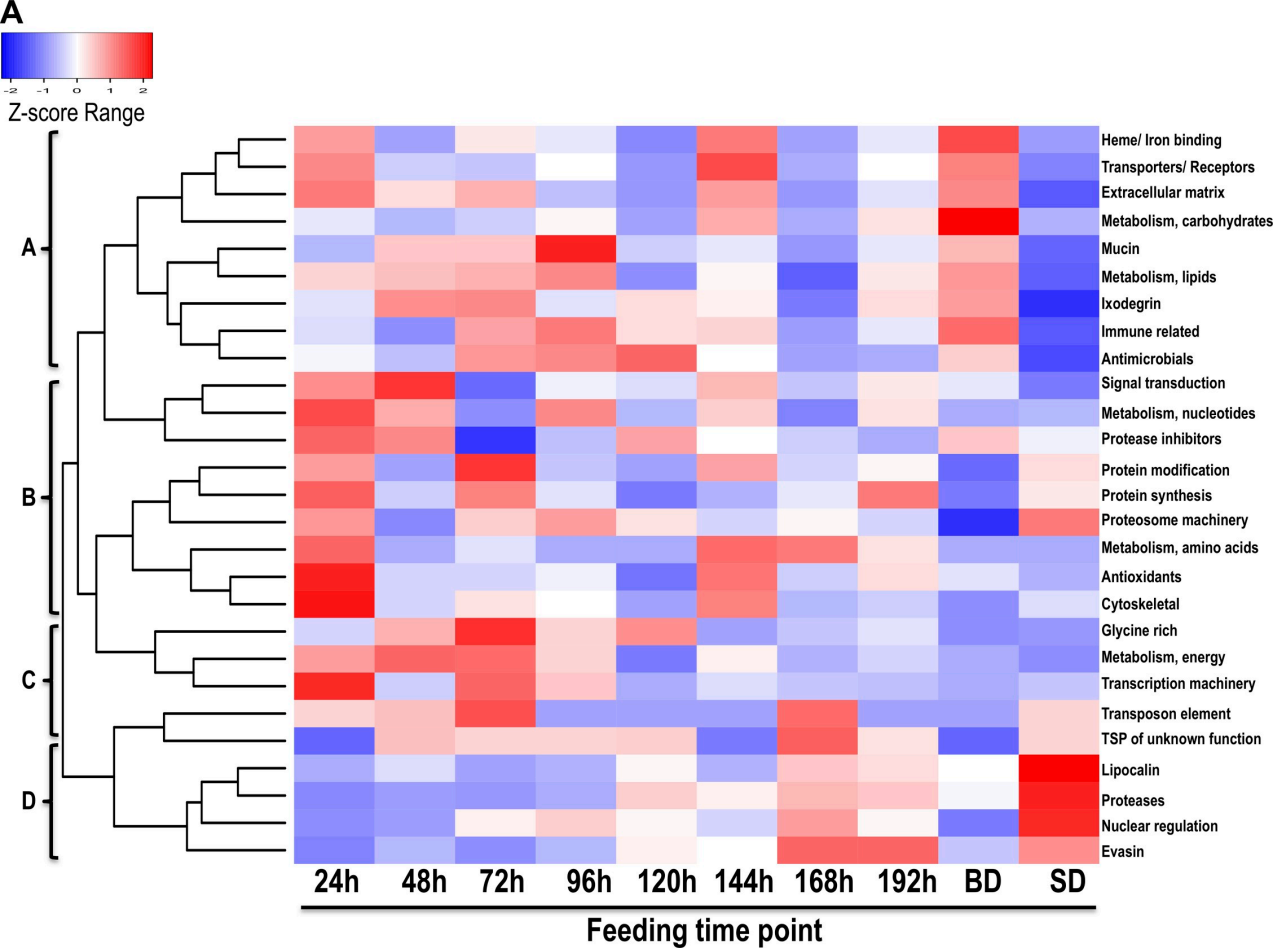
Overall secretion dynamics of tick saliva protein classes in *Amblyomma americanum* tick saliva. Normalized spectral abundance factors (NSAF) values of tick was normalized using the z-score statistics and then used to generate heat maps using heatmap2 function in gplots library using R as described in materials and methods.

**Fig 4:**
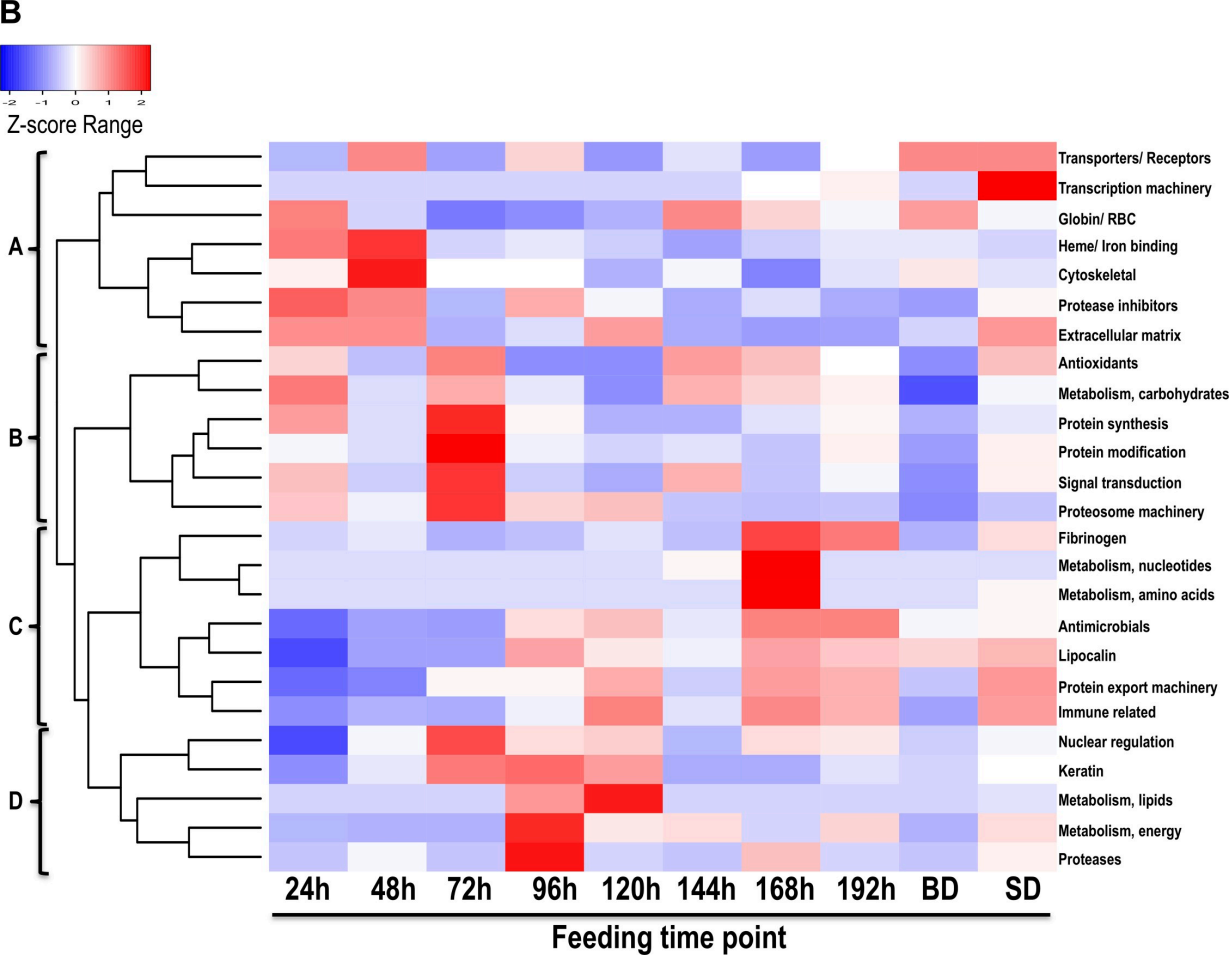
Overall secretion dynamics of rabbit host protein classes in *Amblyomma americanum* tick saliva. Normalized spectral abundance factors (NSAF) values of rabbit host was normalized using the z-score statistics and then used to generate heat maps using heatmap2 function in gplots library using R as described in materials and methods.

To gain insight into broad relationships of secretion dynamics of both tick and rabbit proteins with the tick feeding processes, Z-score statistics normalized NSAF values were visualized on heat maps (Figs 3 and 4). The clustering patterns are influenced by relationships between secretion dynamics of protein category. The blue to red transition denotes low to high abundance. As shown in Fig 3, the 27 tick protein functional categories clustered into four broad secretion patterns (clusters A-D). It is interesting to note that, 74% (20/27) of functional categories are secreted at high abundance within the first 48 h of feeding (Fig 3, clusters A, B, and C) with exception of four categories (evasins, proteases, lipocalins, and nuclear regulation proteins) in cluster D, which are injected into the host at high abundance starting from day five of feeding.

Similarly, the majority of rabbit protein functional categories (21 of the 25) were detected at high abundance in saliva of *A*. *americanum* ticks during feeding (Fig 4). The 25 rabbit protein categories in saliva of *A*. *americanum* ticks segregated into four clusters, A-D (Fig 4). Rabbit proteins that were secreted at high abundance starting from 24–72 h of feeding are part of clusters A and B. Five of the seven proteins in cluster A are highly abundant at 24 and 48 h feeding time points, while those in clusters C and D were less abundant in the first 48 h of feeding and showed varied abundance levels starting from 72 h of feeding.

Ticks initiate the feeding process by secreting an adhesive substance to anchor onto host skin and creating the feeding lesion by lacerating host tissue within the 24–36 h of attachment [62] followed by transmission of some TBD agents after the tick has attached for more than 48 h [63, 64]. On this basis, we speculate that tick proteins in clusters A-C are related with regulating early stages of tick feeding activities associated with initiating tick feeding and regulating transmission of TBD agents. We also speculate that protein categories that were identified in abundance starting from the 192 h feeding time point might be associated with regulating the end of the tick-feeding process, when the tick detaches from the host skin without causing significant damage to host skin.

### Secretion dynamics of non-housekeeping-like *A*. *americanum* tick saliva proteins

S1 Table lists individual proteins that were identified in *A*. *americanum* tick saliva. Thirteen functional categories not considered as housekeeping-like (antimicrobial, detoxification extracellular matrix/cell adhesion, evasin, glycine-rich, heme/iron binding, immunity-related, ixodegrin, lipocalin, mucin, protease inhibitors, proteases, and TSPs of unknown function) (Tables 1 and 2) accounted for 76% of total number of proteins and represented more than 82% in relative abundance throughout feeding time points. In the subsequent sections, we have discussed non-housekeeping-like tick proteins individually per functional category (S1A–S1S Fig) and have highlighted housekeeping-like tick proteins and rabbit proteins as a group below. We have presented and discussed data in S1 Fig. based on similarities in secretion patterns. Please note that the dendrograms were hierarchically clustered based on abundance throughout feeding (using gplot2 software in R). We then manually labeled (with letters) the clusters to discuss the secretion dynamic profiles. Our group is interested and is working to understand functions of proteases and protease inhibitors, and our subsequent discussion below is biased toward these two categories.

### a) *A*. *americanum* tick saliva contains a large diversity of protease inhibitors in nine families

We previously documented at least 18 of the 99 Merops database protease inhibitors (PI) that might be expressed by *A*. *americanum* and other tick species [65]. Here we show that adult *A*. *americanum* ticks secreted at least 155 PIs belonging into eight Merops PI families (S1A–S1E Fig). These include Kunitz-type inhibitors (I2, n = 68), serine protease inhibitors (serpins, I4, n = 21), trypsin inhibitor-like (TIL, I8, n = 36), alpha-2-macroglobulins (α2M, I39, n = 12), cysteine inhibitors (cystatin, I25, n = 12), thyropins (I31, n = 3), phosphatidylethanolamine-binding proteins (I51, n = 2), and a tick carboxypeptidase inhibitor (TCI, n = 1). Of significant interest, nearly 75% of PIs (115/155) in this study were secreted in saliva within the first 120 h of feeding (S1 Table). This strongly suggests that functions of tick saliva PIs are associated with regulating early stages of tick feeding that include tick transmission of TBD agents.

Of the PI families in this study, serpins are the most studied [42, 65–70], presumably because functional roles of this protein category are relatable to tick feeding physiology. To successfully feed and transmit TBD agents, ticks have to overcome serine protease-mediated host defense pathways that are tightly controlled by inhibitors, including serpins. On this basis, it was proposed that ticks might utilize serpins to evade host defenses to successfully feed [71]. From this perspective, it is notable that 90% (19/21) of serpins were identified in saliva of ticks that fed for 24–48 h (S1A Fig), suggesting these serpins are injected into host and might be involved with regulating tick feeding within hours of the tick starting to feed. We would like to note that 5 of the 19 serpins that were identified in saliva of 24–48 h of attachment (Aam-264383, Aam-88534, Aam-264380, Aam-433905, and Aam-1014495) were secreted at high abundance in later feeding time points. It is interesting to note that, *A*. *americanum* serpin 6 (Aam-2673) and 19 (Aam-88534), which were previously validated as inhibitors of host defense system proteases including blood clotting [72, 73] were also found in tick saliva within the first 24 h of feeding this study. Most recently, AAS27 (Aam-28434), that has anti-inflammatory function by inhibiting trypsin and trypsin-like proteases were present in all time points in tick saliva [74]. In another study, we have shown that, AAS41 (Aam-973854), which is secreted at high abundance continuously through 120 h is an inhibitor of inflammation that acts by inhibiting chymase (Kim et al., *in submission*). It is also notable that whereas, 20–50% of all PIs were identified at a single time points, only a single serpin (Aam-264383) was found at a single time point (S1 Table). This might suggest that the functions of tick saliva serpins are important throughout the tick feeding process.

Although not much has been reported on the functional analysis of *A*. *americanum* tick cystatins, a lone study has reported that RNAi-mediated silencing of a cystatin transcript reduced the ability of ticks to feed successfully [75]. Several researchers have reported cystatins in other tick species indicated that they play important roles in tick feeding physiology [76]. In the soft tick *Ornithodoros moubata*, a cystatin was internalized by host dendritic cells and targeted cathepsin S and cathepsin C, affecting their maturation [77]. Cystatins from other tick species also have immunosuppressive functions [78–80]. Of the 12 cystatins identified in tick saliva from this study (S1B Fig, S1 Table), seven were secreted on or after 72 h of feeding, indicating that majority of cystatins might be involved in regulating tick feeding functions after the tick has initiated feeding. On the contrary, five cystatins were secreted within the first 48 h of feeding.

Similar to cystatins, S1C Fig shows the secretion dynamics of alpha-2-macroglobulins (α2M), where less than half (4/12) were injected into the host at high abundance at start of feeding (24–48 h), and another two at the 96 h feeding time point (clusters A and B). It is also notable that the remaining 6 of 12 were injected at high abundance at the 144 h time point (clusters C and D). This might suggest that α2M could be involved in regulating tick feeding functions toward the end; we normally observe ticks starting to engorge and complete feeding after 120 h of attachment. There are very few studies on α2M in tick feeding physiology. Two studies have reported involvement of α2M in immune defense of soft ticks [81] and anti-microbial activity in *I*. *ricinus* [82].

Kunitz-type inhibitors and trypsin inhibitor-like (TIL) were the majority of PIs that were found in this study. The secretion dynamics of Kunitz-type inhibitors (S1D Fig) and TIL (S1E Fig) is comparable and notable: the tick appears to secrete a different set of these inhibitors every 24 h starting from the first day of tick feeding. This might suggest that functions of these inhibitors are required throughout the tick feeding process. The observed alternate secretion pattern might signal the potential for ticks to evade host defense in that host immunity against 24 h secreted inhibitors might not be effective against a different set that is secreted at subsequent time points. It is also notable that a total 35% of Kunitz-type inhibitors and 22% of TILs were detected in saliva of replete fed ticks (SD), unlike the other PIs in this study, suggesting a role toward end of tick feeding.

### b) Majority of proteases in *A*. *americanum* saliva are metalloproteases

At the time of this study, protease families that were encoded by *A*. *americanum* were not enumerated, presumably because its genome has not been sequenced. However, analysis of annotated sequences from *I*. *scapularis* showed that the tick might encode for all protease categories: aspartic, cysteine, serine, metallo-, and threonine proteases [83]. Here, we found that *A*. *americanum* secretes at least 94 proteases in saliva during feeding. These 94 proteases belong in four categories grouped into 15 families: aspartic (family A1, n = 4), cysteine (C1, C2, and C13, n = 12), metallo- (M12, M13, M14, M15, M17, M20, M28, and M49, n = 56), and serine (S1, S10, and S28, n = 22) proteases (S1 Table, S1F, S1G and S1H Fig). Please note that the heatmap for aspartic proteases was not developed due to low numbers (the secretion dynamics is presented in S1 Table). The heatmaps (S1F, S1G and S1H Fig and S1 Table) show that more than 60% (60/94) of proteases are injected into the host at various time points during the first five days of feeding, demonstrating that some of the proteases in this study are associated with regulation of initial tick feeding processes.

The observation that metalloproteases are the majority of proteases in saliva of *A*. *americanum* is consistent with our previous findings in the *I*. *scapularis* proteome [32]. It is notable that similar to the *I*. *scapularis* proteome, metalloproteases that were secreted at high abundance during the first 72 h feeding time points are in families M12 and M13 (S1G Fig), indicating that these proteases regulate initial tick feeding functions that are important to both tick species. Indirect evidence on snake venom M12 proteases that have anti-coagulant activity [84, 85] suggest that secretion of these proteases at high abundance when the tick is initiating feeding might be beneficial to the tick as they might function to prevent blood from clotting. There is also evidence that RNAi-mediated silencing of M12 proteases significantly affected tick-feeding efficiency [86]. There is evidence that *I*. *scapularis* secretes a metalloprotease that is similar to snake venom hemorrhagic proteases that degrades gelatin, fibrin(ogen), and fibronectin [87]. Likewise, indirect evidence suggests that ticks might utilize M13 proteases to regulate host immunity. In mammals, M13 proteases were among other functions, involved in modulating neurotransmitter levels, control blood pressure, involved in reproduction and cancer progression [88].

Another notable similarity between *A*. *americanum* and *I*. *scapularis* proteomes is that both tick species secreted a small number of S1 serine proteases, six and three respectively (S1G Fig and S1 Table). We are interested in S1 serine proteases due to their functional roles in signal transduction as activators of protease-activated receptors [89, 90]. Therefore, it is potentially possible that the tick utilizes these proteases to interfere with host defense signaling at the tick-feeding site.

The observation that *A*. *americanum* injected cysteine proteases at the beginning of feeding indicate they might be playing some role(s) in the early stages of tick feeding. Several studies have documented potential functional roles of cysteine proteases in tick physiology [91–93]. In a lone study, a cysteine protease from *H*. *longicornis* when silenced by RNAi, showed to be involved with digestion of a blood meal and increased the number of *Babesia* parasites [94]. Recently, a cathepsin L from the tick, *R*. *microplus* (BmCL1), was shown to interact with thrombin at pH 7.5 and impair thrombin-induced fibrinogen clotting via a fibrinogenolytic activity [95]. In helminths, cysteine proteases are the most abundant category of proteins identified into excretion/secretion products [96] and have been shown to be involved with host immune evasion [97] and extracellular matrix degradation [98].

Majority of studies on tick aspartic proteases are mainly characterized as blood digestion proteins in the midgut, similar to the mammalian lysosome acidic protease, cathepsin D [99]. In *H*. *longicornis* adult ticks, the potential role of these proteins in proteolysis of erythrocyte hemoglobin has been reported [100]. Other studies have shown the importance of this protease in embryogenesis, playing roles in vitellin degradation [101] and heme-binding properties [102]. Although only four aspartic proteases were identified in *A*. *americanum* saliva during feeding, three of these proteases were present within the first 96 h of feeding, which may implicate roles in the early stages of tick feeding success (S1 Table).

### c) Lipocalins/histamine-binding proteins are alternately secreted during tick feeding

Inflammation response is among host defense pathways that ticks must evade to complete feeding. Histamine is one of the key mediators of inflammation in tissue damage that is expected to occur in response to tick feeding [103]. From this perspective, lipocalins/tick histamine-binding proteins in tick saliva are suspected to be part of the tick machinery to evade the host’s inflammation defense response through sequestration of histamine that is released at the tick-feeding site. In this study, we found 46 lipocalins/tick histamine-binding proteins that show two broad secretion patterns: secreted at multiple feeding time points and those that were alternately secreted at single time points (S1I Fig). It is interesting to note that of the total 46 lipocalins identified in tick saliva during feeding, 22% (10/46) were present within the first 48 h of feeding, while 35% (16/46) were present after 96 h of feeding, and 43% (20/46) were identified in a single time point (S1I Fig, S1 Table). Given that in addition to regulating inflammation, lipocalins/histamine-binding proteins have other diverse functions such as antimicrobials [104, 105], glucose metabolism [106] and binding several ligands including serotonin and fatty acids [107, 108], it is most likely that these proteins might be involved in regulating several other tick feeding functions besides mediating the tick’s anti-inflammation function.

### d) Heme binding proteins are secreted at high abundance throughout feeding

Like other animals, ticks require iron and heme (the iron-containing part of hemoglobin) for normal physiological functions [109]. However, ticks do not have a heme biosynthesis pathway, therefore they must obtain it from host blood [110]. Female ticks that were artificially fed a diet not containing hemoglobin laid sterile eggs [111] demonstrating the importance of heme in tick biology. However, in high abundance heme can be toxic for the tick [112], therefore it is postulated that hemelipoproteins and vitellogenenins could serve as heme binding proteins to remove the excess heme from the tick system. S1J Fig and S1 Table lists a total of 17 heme/iron-binding proteins consisted of hemelipoproteins, vitellogenins, and a ferritin that collectively accounted for the third most abundant protein category in tick saliva throughout feeding. High abundance of hemelipoproteins here is in consistent with other tick saliva proteomes [32–34]. The secretion dynamics summarized in S1J Fig revealed two broad secretion patterns, those that are injected into the host from 24 h through 120 h of feeding (HCB and HCC) and those that are injected into the host starting from 144 h of feeding through the end of tick feeding (HCD and HCA). Ticks acquire both iron and heme from host blood [110, 113], and thus iron and/heme-binding proteins are important to normal tick physiology. It has been shown that *R*. *microplus* hemelipoprotein (HeLp) could bind eight heme molecules [114]. Given that hemelipoprotein is the most abundant protein in tick hemolymph [115], it could be secreted in saliva as a result of this protein being transferred into the salivary glands. There is also evidence that mRNA of hemelipoproteins is also expressed in salivary glands [116] of unfed and fed adult ticks and might suggest that these proteins could have different functions during blood meal feeding by ticks. It is also known that free heme has pro-inflammatory properties [117]. Thus, the presence of hemelipoproteins could lower free heme concentration at the feeding site, and as a result reduce the potential for heme to promote inflammation. Indirect evidence suggest that tick saliva hemelipoproteins might also serve as antioxidants and transporters of other compounds such as cholesterol, phospholipids, and free fatty acids [118]. The role of vitellogenin-like proteins in tick feeding remain to be established. It is interesting to note that although vitellogenin is predominant, reduction of vitellogenin receptor (VgR) expression by RNAi-mediated silencing resulted in reduced fertility [119] and *Babesia bovis* transmission and oocyte maturation [120].

### e) Ticks inject multiple antioxidant proteins into the feeding site

Feeding and digestion of large amounts of host blood exposes ticks to hydroxyl radicals and reactive oxygen species (ROS), which if left uncontrolled could damage tick tissue [121, 122]. Expression of antioxidant proteins protect the tick during feeding and digestion of the blood meal. Studies have shown that RNAi-mediated silencing of tick antioxidants caused deleterious effects to the tick and prevented them to obtain a full blood meal [123, 124]. Previous studies by others and from our group have documented presence of antioxidants in tick salivary glands [125, 126] and saliva [32–34, 127, 128]. In this study we identified 41 putative antioxidant enzymes. These enzymes include glutathione-S-transferase, thioredoxin, superoxide dismutase, catalase, peroxinectin, arylsulfatase, aldehyde dehydrogenase, epoxide hydrolase, sulfotransferase, sulfhydryl oxidase and glycolate oxidase. S1K Fig reveals two broad secretion patterns of tick saliva antioxidant proteins based on NSAF values as an index for abundance: (i) proteins injected into the host in high abundance once at various feeding time points (ACA-ACI) and (ii) proteins that are consecutively injected into the host in high abundance starting from 24–96 h of feeding (ACF). Like heme/iron binding proteins, tick antioxidants are presumed to function inside the tick and the role(s) these proteins in the feeding regulation remain to be defined. We speculate that as host tissue injury caused during the creation of the tick-feeding site could trigger release of oxidants such as ROS, some of the tick saliva antioxidants might function to counter oxidant molecules to protect tick tissue.

### f) Glycine-rich and extracellular matrix/cell adhesion proteins are secreted early during tick feeding

Within 5–30 min of attachment, the tick secretes an adhesive substance called cement, which anchors ticks onto host skin during its protracted feeding period [62]. Tick cement is also suggested to protect the tick from host immune factors [129, 130] and might function as antimicrobials at the feeding site [131]. Glycine-rich proteins are among categories of tick proteins that are thought to play key roles in formation of tick cement [62]. From this perspective, glycine-rich proteins are among tick proteins that have received significant research attention [132–135]. In this study we found a total of 67 glycine-rich proteins, which represented the fifth largest category of proteins identified in tick saliva during feeding (Fig 1, S1 Table). Nearly 90% (60/67) of the glycine-rich proteins were secreted in abundance within the first four days of feeding (S1L Fig; GCB, GCD, GCE, GCF, and GCG). Tick cement deposition is completed during the first 96 h of tick feeding [62], and thus it is conceivable that some of the glycine-rich proteins in this study might be involved with tick cement formation. It is interesting to note that some of the glycine-rich proteins that were identified from tick cement in our group [134] and others [133] were also found in this study (S2 Table). Some of the glycine-rich proteins were secreted from the 144 h time point, long after tick cement formation; these might regulate other tick feeding functions. Although glycine-rich proteins are mostly known for their potential role in tick cement formation, indirect evidence in other organisms indicate that these proteins might be involved in other functions such as host defense and stress response as in plants [136].

S1M Fig. summarize the secretion dynamics of 37 extracellular matrix proteins that were found in this study. Similar to glycine-rich proteins, majority (27/37) of the extracellular proteins were secreted within the first five days of feeding demonstrating their role in early stage tick feeding regulation. Our speculation is that some of these proteins will play roles in formation of tick cement. In a previous study, RNAi-mediated silencing of chitinase, also identified in this study, weakened the tick cement cone to the extent that host blood was leaking out around the mouthparts of attached ticks [40].

### g) Antimicrobials, mucins, and immune related proteins are secreted throughout the feeding process

Once the tick has anchored itself onto the host skin and created its feeding lesion, it faces a difficult task of overcoming host humoral and cellular immunity, and also preventing microbes in the host skin from colonizing the tick-feeding site. Here we show that *A*. *americanum* secretes immunomodulatory and antimicrobial peptides starting within the early stages of the tick feeding process (S1N–S1R Fig). We identified nine antimicrobials consisting of microplusins, lysozymes, and defensins (S1N Fig). Previous studies showed that microplusin has dual effects against fungus and gram-positive bacteria, lysozyme against gram-positive bacteria, and defensin effective against both gram-positive and -negative bacteria [137–139]. The heat map in S1N Fig shows that antimicrobials were injected into the host starting at 24 and 48 h (AMCA), from 72 h (AMCC), and from 120 h (AMCB). This secretion pattern suggests that the functions of antimicrobial peptides are needed throughout feeding.

Similar to antimicrobials, we identified 12 mucins (S1O Fig), with ~60% of these proteins (7/12) being secreted at high abundance within 24–48 h of feeding. Functional roles of mucins in ticks have not been studied. However, indirect evidence in mammals suggest that mucins might be involved in antimicrobial activity in that human mucins were shown to encapsulate microbes [140].

Among putative immunomodulatory proteins, we identified evasins (S1P Fig) and ixodegrins (S1Q Fig). Evasins (n = 12, S1P Fig) were shown to bind to chemokines [141, 142] to reduce leukocytes recruitment to the tick feeding site and therefore contribute to tick evasion of the host’s inflammatory defense. It is interesting to note that the 12 evasins identified in tick saliva were present after 24 h of feeding and continued to be secreted throughout feeding at variable levels. This might suggest that evasins might not be involved in regulating tick feeding functions during the first 24 h of tick feeding.

S1Q Fig summarizes the secretion pattern of the six ixodegrin-like proteins found in tick saliva during feeding in this study. It is interesting to note, 83% (5/6) of these proteins were identified within the first 48 h of feeding (S1 Table). These proteins were first described in *I*. *scapularis* as inhibitors of platelet aggregation [143]. Platelet aggregation is the first step in the blood clotting system [144], which ticks must overcome to successfully feed. Thus, the presence of ixodegrins in saliva of *A*. *americanum* at the start of feeding is beneficial to tick feeding success. Finally, we also found proteins that show similarity to previously characterized immunomodulatory proteins (S1R Fig), which have been validated in other tick species including p36, which inhibits cell proliferation and cytokine expression [145]. These proteins might play roles in mediating the tick’s evasion of host immunity.

### h) Tick-specific secreted saliva proteins (TSP) of unknown function are alternately secreted

Over one-third of Ixodidae protein sequences deposited into GenBank are annotated as hypothetical, secreted, conserved and unknown proteins. However, some are annotated based on sequence identities and conserved signature motifs, which include basic tail/tailless proteins, 8.9 kDa protein family, leucine-rich proteins, AV422 (a tick saliva protein that is high upregulated when ticks are stimulated to start feeding [39, 146]), proteins containing RGD motifs, which might play roles in inhibition of platelet aggregation [143, 147]. In this study we have identified a total of 377 (S1S Fig) tick saliva proteins that fit the above description that we refer here to as tick-specific saliva proteins of unknown functions (TSPs). More than 95% (357/377) of the total TSPs were identified within the first eight days of feeding in tick saliva indicating their potential roles in regulating the tick feeding process. It is interesting to note that the secretion pattern for over a third (128/377) of the total TSPs identified in tick saliva during feeding were alternately injected once during feeding (S1 Table). From the perspective of finding target antigens for tick vaccine development, TSPs represent a unique opportunity in that they do not share any homology to host proteins and might not cross-react with the host.

### *A*. *americanum* secretes multiple housekeeping-like proteins in saliva throughout the feeding process

S1 Table lists 288 housekeeping-like proteins that were identified in this study. Presence of these proteins in *A*. *americanum* saliva is not unexpected, as similar findings have been previously reported in tick saliva [32–34]. The 288 housekeeping-like proteins were classified into 14 categories including those associated with metabolism of amino acids (n = 7), carbohydrates (n = 25), energy (n = 31), lipids (n = 31), and nucleic acids (n = 33). Other protein categories include those involved in cytoskeletal (n = 53), nuclear regulation (n = 16), protein modification (n = 21), proteasome machinery (n = 8), protein synthesis (n = 10), signal transduction (n = 24), transposable element (n = 3), transcription machinery (n = 7), and transporter/receptors (n = 17). It is interesting to note that, within the first 24 h of feeding 12 of the 14 categories were identified at high abundance (S1A Fig).

One feature of housekeeping-like tick proteins is that they have high sequence identity with mammalian housekeeping proteins, and for this reason they are discounted as potential target antigens for tick vaccine development. However, based on literature showing that several roles of these proteins in host defense, we think that these proteins play an important role in tick feeding physiology. Housekeeping-like proteins identified here mostly function intracellularly, and they serve as alarm signals to alert the host defense system to injury when secreted outside of the cell [148]. There is evidence that in the extracellular space, some of the housekeeping proteins such as heat shock proteins, have anti-inflammatory functions [149], while histone proteins have antimicrobial activity [150]. Given high sequence similarity to host housekeeping proteins, it is possible that some of the tick housekeeping-like proteins play roles in promoting tick feeding through anti-inflammatory and anti-microbial activity.

Another important aspect of tick feeding physiology that has not received much attention is the fact that host blood meal also contains carbohydrates, lipids and other molecules besides host proteins. It is notable that some of the tick housekeeping-like proteins in tick saliva have high similarity to enzymes in the carbohydrate and lipid metabolism pathways. It will be interesting to determine if injecting these proteins into the feeding site helps the tick to break down host blood carbohydrates and lipids into units that can be easily taken and assimilated by ticks.

### Secretion of rabbit host proteins in *A*. *americanum* tick saliva is not random

In this study, we identified 335 rabbit host proteins belonging into 25 different functional categories that include cytoskeletal (19%), keratin (13%), nuclear regulation (8%), immune-related (8%), hemoglobin/RBC degradation (6%), transporters/receptors (5%), protein modification (5%), and protein categories below 4% included antimicrobials, extracellular matrix, heme/iron binding, detoxification/ antioxidants, metabolism (energy, carbohydrates, lipid, amino acid, and nucleic acids), protein export, protein synthesis, fibrinogen, protease inhibitors, proteases, signal transduction, transcription machinery, proteasome machinery, and lipocalin (Tables 3 and 4, S1 Table). Relative abundance as determined by NSAF indicated that the most abundant protein categories consisted of hemoglobin/RBC degradation products (58–13%), followed by heme/ iron binding host proteins (13–16%), and cytoskeletal (6–20%) (Fig 4).

At a glance, presence of rabbit host proteins in *A*. *americanum* tick saliva could be dismissed as host protein contamination. This observation might be strengthened by the fact that some rabbit host proteins in tick saliva such as keratin, nuclear regulation proteins, and host antimicrobial peptides increased in abundance as feeding progressed. This suggested that secretion of host proteins into tick saliva was a consequence of ticks ingesting an increased amount of host blood, and that some of these host proteins might leak or be regurgitated back into the host via saliva or esophagus. However, our data here suggests that the tick might systematically be utilizing host proteins to regulate its tick-feeding site. For instance, mammals are likely to encode for more than 500 proteases and 150 protease inhibitors (based on rat, mice, and humans [151]), however we found 9 proteases and 8 protease inhibitors from host origin in *A*. *americanum* tick saliva (S1 Table). We are of the view that if secretion of host proteins was a random process, we could have identified more rabbit host proteases and protease inhibitors. There are reports that human α1-antitrypsin and α2-macroglobulin are secreted following injury as occurs during tick feeding, and if left uncontrolled could lead to delayed wound healing [152], which is beneficial to tick feeding. On this basis, it is highly likely that ticks inject host α1-antitrypsin and α2-macroglobulin into the feeding site as a strategy of evading the host’s tissue repair defense response. It is also notable that fibrinogen and neutrophil gelantinase-associated lipocalin, which among other functions plays important roles in wound healing, were identified towards the end of feeding [153–156]. This is interesting in that the tick-feeding lesion is completely sealed, preventing leakage of blood, when a replete fed tick detaches from its feeding site. It has been reported in *Opisthorchis viverrini*, the human liver fluke, that they secrete proteins in the granulin family that accelerate wound healing [157]. It is possible that the increased abundance of host proteins involved in wound healing are secreted by the tick into the feeding site towards the end of tick feeding is the tick’s way to help its host heal.

### Different tick species might utilize similar proteins to regulate feeding

At the time of preparing data in this study for publication, several other tick saliva proteomes had been published. We took advantage of the availability of these data to test the hypothesis that key proteins that are important to tick feeding might be conserved across tick taxa. Thus, we compared data in this study to saliva proteomes of *I*. *scapularis* [32], *R*. *microplus* [33], *H*. *longicornis* [34], *R*. *sanguineus* [35], *D*. *andersoni* [36], and *O*. *moubata* [37]. This analysis revealed that more than 24% (284/1182) of the *A*. *americanum* tick saliva proteins in this study have homologs that were secreted in saliva of other tick species (S3 Table). Table 5 highlights the 163, 138, 137, 92, 22, and 11 *A*. *americanum* tick saliva proteins in 22 functional categories that were >70% identical to proteins in saliva of *I*. *scapularis* [32], *H*. *longicornis* [34], *D*. *andersoni* [36], *R*. *microplus* [33], *O*. *moubata* [37] and *R*. *sanguineus* [35], respectively.

**Table 5:** *Amblyomma americanum* tick saliva protein categories that are conserved in other tick saliva proteomes at 70% identity.

Classification	*I*. *scapularis*	*H*. *longicornis*	*D*. *andersoni*	*R*. *microplus*	*O*. *moubata*	*R*. *sanguinues*
Cytoskeletal	36	27	19	12	3	0
Detoxification	13	9	9	5	0	2
Extracellular matrix	3	6	9	3	0	1
Glycine rich	5	4	4	4	0	0
Immune related	4	3	3	4	1	1
Metabolism, amino acids	4	1	0	0	0	0
Metaolism, carbohydrates	4	3	6	1	1	0
Metabolim, energy	20	11	12	0	4	0
Metabolism, lipids	2	1	3	4	0	0
Metabolism, nucleic acids	11	9	1	0	2	0
Nuclear regulation	6	5	6	4	0	0
Protein modification	16	14	8	9	7	0
Protease	6	6	8	3	0	0
Proteosome machinery	7	6	5	0	0	6
Protein synthesis	4	2	3	2	0	0
Secreted saliva proteins	5	6	22	14	0	0
Lipocalin	0	0	0	4	0	0
Protease Inhibitors	5	14	10	11	1	1
Signal transduction	6	3	6	1	1	0
Heme/Iron binding	1	7	2	9	2	0
Transcription machinery	2	0	1	1	0	0
Transporters/ receptors	3	1	0	1	0	0
Total Protein Matches	163	138	137	92	22	11

Of the 22 categories of proteins, immune-related proteins were present in all tick saliva proteomes. Likewise, proteins from nine other categories (antioxidant/detoxification, carbohydrate metabolism, cytoskeletal, extracellular matrix, heme/iron binding protease, protease inhibitor, protein modification, and signal transduction) from *A*. *americanum* saliva were present in saliva of five other tick species. It is notable that tick-specific proteins were the highest conserved among tick species with the exception of *R*. *sanguineus*, for which limited data is available. It is notable that five *A*. *americanum* TSPs were highly conserved in *I*. *scapularis*, a Prostriata tick, and 6, 22, and 14 in Metastriata ticks; *H*. *longicornis*, *D*. *andersoni*, and *R*. *microplus*, respectively.

Of the 22 categories of proteins, immune-related proteins were present in all tick saliva proteomes. Likewise, proteins from nine other categories (antioxidant/detoxification, carbohydrate metabolism, cytoskeletal, extracellular matrix, heme/iron binding protease, protease inhibitor, protein modification, and signal transduction) from *A*. *americanum* saliva were present in saliva of five other tick species. It is notable that tick-specific proteins were the highest conserved among tick species with the exception of *R*. *sanguineus*, for which limited data is available. It is notable that five *A*. *americanum* TSPs were highly conserved in *I*. *scapularis*, a Prostriata tick, and 6, 22, and 14 in Metastriata ticks; *H*. *longicornis*, *D*. *andersoni*, and *R*. *microplus*, respectively.

We would like the reader to note that with the exception of *I*. *scapularis* tick saliva proteome for which proteins were identified every 24 h during feeding [32] the other tick saliva proteomes were limited to a narrow range of tick feeding time points and/or fully engorged ticks. This might be the reason that higher numbers of tick saliva proteins were conserved between *A*. *americanum* and *I*. *scapularis* tick saliva proteome. It is interesting to note that, *A*. *americanum* and *I*. *scapularis* are biologically different as they belong to different tick lineages, Prostriata and Metastriata [158, 159]. Thus, tick saliva proteins that are shared between these two tick species could regulate evolutionarily conserved proteins essential for tick feeding physiology functions. On this basis, such proteins could be targeted for tick vaccine development. We have previously shown that RNAi-mediated silencing of *A*. *americanum* tick saliva serpin 19, an anti-coagulant, [73], which is also conserved in *I*. *scapularis* ticks [42, 65], caused significant mortality demonstrating the importance of this protein in tick physiology.

## Conclusion and future perspective

This study has made a unique contribution toward understanding the molecular basis of *A*. *americanum* tick feeding physiology. We believe that this study provides a good starting point toward discovery of effective targets for anti-tick vaccine development. Our strategy to identify tick saliva proteins every 24 h during feeding has allowed us to map tick saliva proteins to different phases of the tick feeding process. This is significant as it provides for the opportunity to focus on tick saliva proteins that regulate the tick feeding process that precede critical events such as TBD agent transmission. Majority of TBD agents are transmitted after 48 h of tick attachment [63, 64], and therefore proteins that are secreted from 24 and 48 h of tick feeding time points are prime candidates for tick vaccine research. It is important to acknowledge the fact that, during the course of feeding, *A*. *americanum* ticks secretion of more than 1500 tick and rabbit host proteins might indicate that the tick has inbuilt systems to evade host immunity, and that it is going to be a challenge to actually find effective targets for anti-tick vaccine development. However, the findings that nearly 300 *A*. *americanum* tick saliva proteins were also secreted by other tick species is very encouraging as these proteins might provide insight into conserved mechanisms that are utilized by all ticks to successfully feed and could serve as potential targets for anti-tick vaccine development.

We have recently described proteins (n = 340) in saliva of unfed *A*. *americanum* ticks that were stimulated to start feeding on three different hosts: rabbits, dogs, and humans [38]. It is notable that 70% (231/340) of proteins in saliva of unfed *A*. *americanum* ticks were found in the tick saliva proteome described here (S4 Table). The significance of these data is that the 231 tick saliva proteins present in saliva of both unfed and fed ticks represent proteins that are potentially injected into the host within minutes of the tick attaching onto host skin and are likely associated with regulating initial tick feeding events. Immunologically blocking functions of these proteins might significantly disrupt tick feeding and prevent transmission of TBD agents. In summary, this study has set the foundation for in-depth studies to understand *A*. *americanum* tick feeding physiology and find effective targets for development of tick-antigen based vaccines to prevent TBD infections. It is important to note here that, while this study has provided a valuable starting point in discovery of anti-tick vaccine antigens, the next phase of the research to define the anti-tick vaccine efficacy is the most critical.

## Supporting information

S1 Fig Secretion dynamics of non-housekeeping tick saliva protein families in *Amblyomma americanum* tick saliva. Normalized spectral abundance factors (NSAF) values of tick saliva proteins that did not show similarity to housekeeping proteins were normalized using the z-score statistics and then used to generate heat maps using heatmap2 function in gplots library using R as described in materials and methods. (Protease Inhibitors are labeled as A- Serpins, B- Cystatins, C- ⍺2-macroglobulin, D-Kunitz type, E- trypsin inhibitor like; Protease are labeled as F- cysteine, G- metalloprotease, H- serine; and other protein classes as I- Lipocalin, J- heme/iron binding, K- antioxidants, L- glycine rich, M- extracellular matrix, N- antimicrobial, O- Mucin/ mucin-like, P- Evasin, Q- Ixodegrin, R- Immune related, and S- tick specific secreted saliva proteins of unknown function).
(TIFF)

S1 Table List of identified *A. americanum* tick, rabbit, contaminant, and reversed sequence proteins in tick saliva during feeding using LC-MS/MS. The peptide count, spectral count, normalized spectral abundance factor (NSAF), exponentially modified protein abundance index (EMPAI), spectral count, and sequence coverage (%) generated from the Prolucid and IDcompare analyses are represented on an excel file with different tablatures for tick, rabbit host, contaminants, and reversed-sequences. The time point during tick feeding is noted for every 24 h, BD represents manually detached ticks that were apparently fully engorged but not yet detached, and SD represents spontaneously detached fully engorged ticks. The contig represents the identifier for the CDS extracted from the assembled BioProject accession # PRJNA226980. The description is the nomenclature of the putative protein, the classification represents the proteins functional category, occurrence represents the number of times the peptides matching to putative proteins were identified using LC-MS/MS during feeding time points, and status is a binary representation of when the peptides matching to putative proteins were detected (1) or not (0) during feeding time points.
(XLSX)

S2 Table *A. americanum* saliva proteins during feeding matches to published cement proteomes. *A*. *americanum* tick saliva proteins identified using LC-MS/MS from this study were compared to currently published *A*. *americanum* ticks saliva proteomes. The peptide count, spectral count, normalized spectral abundance factor (NSAF), exponentially modified protein abundance index (EMPAI), spectral count, and sequence coverage (%) generated from the Prolucid and IDcompare analyses are represented on an excel file. The time point during tick feeding is noted for every 24 h, BD represents manually detached ticks that were apparently fully engorged but not yet detached, and SD represents spontaneously detached fully engorged ticks. The identifier represents the contig for the CDS extracted from the assembled BioProject accession # PRJNA226980, or from NCBI GenBank accession numbers. Identifiers with no matches were noted with N/A. The description is the nomenclature of the putative protein, the classification represents the proteins functional category, occurrence represents the number of times the peptides matching to putative proteins were identified using LC-MS/MS during feeding time points, and status is a binary representation of when the peptides matching to putative proteins were detected (1) or not (0) during feeding time points.
(XLSX)

S3 Table *A. americanum* saliva protein matches (>70% identity) to other tick saliva proteomes. *A*. *americanum* tick saliva proteins identified using LC-MS/MS from this study were compared to currently published tick saliva proteomes of *I*. *scapularis*, *H*. *longicornis*, *R*. *microplus*, *D*. *andersoni*, *O*. *moubata*, and *R*. *sanguineus*. Contig numbers are noted for *A*. *mericanum* proteins that matched (+) or not matched (-) to other tick saliva proteomes. The description is the nomenclature of the putative protein, the classification represents the proteins functional category, occurrence represents the number of times the peptides matching to putative proteins were identified using LC-MS/MS when compared to other tick saliva proteomes, and status is a binary representation of when the peptides matching to putative proteins were detected (1) or not (0) when comparing to other tick saliva proteomes.
(XLSX)

S4 Table *A. americanum* saliva proteins during feeding present in unfed *A. americanum* saliva proteome. *A*. *americanum* tick saliva proteins identified using LC-MS/MS from this study were compared to *A*. *americanum* unfed saliva proteome. Contig numbers are noted for *A*. *mericanum* proteins from this study and from Tirloni et al., (2017) that were present in both proteomes. The description is the nomenclature of the putative protein, the classification represents the proteins functional category, occurrence represents the number of times the peptides matching to putative proteins were identified using LC-MS/MS in unfed non stimulated, host stimulated (dog, human or rabbit), or fed stages every 24 h, and status is a binary representation of when the peptides matching to putative proteins were detected (1) or not (0) in unfed non stimulated, host stimulated (dog, human or rabbit), or fed stages every 24 h. BD represents manually detached ticks that were apparently fully engorged but not yet detached, and SD represents spontaneously detached fully engorged ticks.
(XLSX)
