# Supplementary figures and images for: Time-resolved proteomic profile of Amblyomma americanum tick saliva during feeding
Source: PLoS Negl Trop Dis. 2020 Feb 12;14(2):e0007758. doi: 10.1371/journal.pntd.0007758 (PMC7041860; doi:10.1371/journal.pntd.0007758)

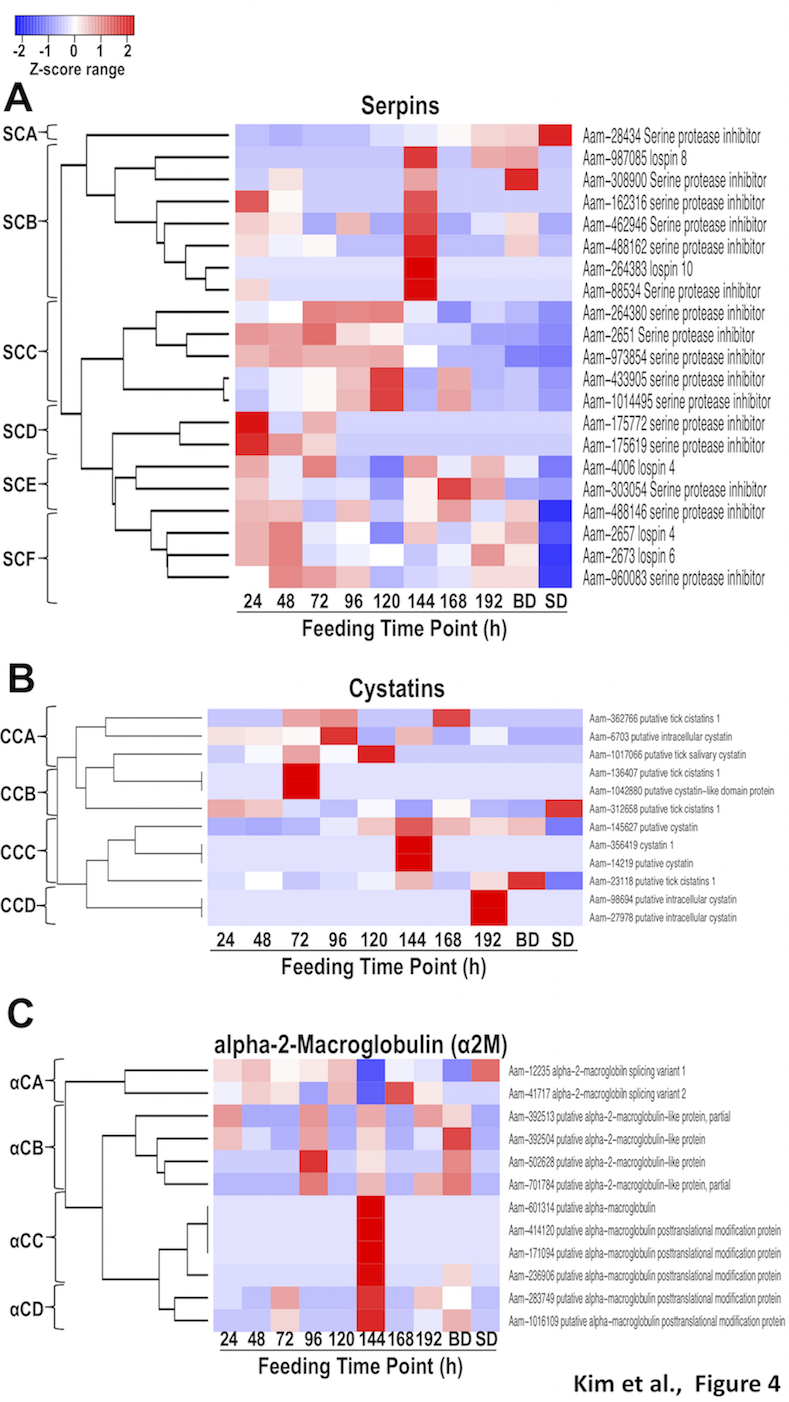

Supplement: S1 Fig — Normalized spectral abundance factors (NSAF) values of tick saliva proteins that did not show similarity to housekeeping proteins were normalized using the z-score statistics and then used to generate heat maps using heatmap2 function in gplots library using R as described in materials and methods. (Protease Inhibitors are labeled as A- Serpins, B- Cystatins, C- ⍺2-macroglobulin, D-Kunitz type, E- trypsin inhibitor like; Protease are labeled as F- cysteine, G- metalloprotease, H- serine; and other protein classes as I- Lipocalin, J- heme/iron binding, K- antioxidants, L- glycine rich, M- extracellular matrix, N- antimicrobial, O- Mucin/ mucin-like, P- Evasin, Q- Ixodegrin, R- Immune related, and S- tick specific secreted saliva proteins of unknown function). (TIFF) [file pntd.0007758.s001.tiff]
